# Coagulation competence for predicting perioperative hemorrhage in patients treated with lactated Ringer’s vs. Dextran - a randomized controlled trial

**DOI:** 10.1186/s12871-015-0162-1

**Published:** 2015-12-08

**Authors:** Kirsten C. Rasmussen, Michael Hoejskov, Per I. Johansson, Irina Kridina, Thomas Kistorp, Lisbeth Salling, Henning B. Nielsen, Birgitte Ruhnau, Tom Pedersen, Niels H. Secher

**Affiliations:** 1 Departments of Anesthesiology, Transfusion Medicine, University of Copenhagen, Copenhagen, Denmark; 2 Departments of Urology and Centre for Head and Orthopaedic Surgery, Rigshospitalet, University of Copenhagen, Copenhagen, Denmark; 3 Department of Anesthesiology, Rigshospitalet 2043, Blegdamsvej 9, DK-2100 Copenhagen Ø, Denmark

**Keywords:** Coagulation, Colloid, Crystalloid, Fluid therapy, Hemorrhage

## Abstract

**Background:**

Perioperative hemorrhage may depend on coagulation competence and this study evaluated the influence of coagulation competence on blood loss during cystectomy due to bladder cancer.

**Methods:**

Forty patients undergoing radical cystectomy were included in a randomized controlled trial to receive either lactated Ringer’s solution or Dextran 70 (Macrodex ®) that affects coagulation competence.

**Results:**

By thrombelastography evaluated coagulation competence, Dextran 70 reduced “maximal amplitude” (MA) by 25 % versus a 1 % reduction with the administration of lactated Ringer’s solution (*P* <0.001). Blinded evaluation of the blood loss was similar in the two groups of patients - 2339 ml with the use of Dextran 70 and 1822 ml in the lactated Ringer’s group (*P* = 0.27). Yet, the blood loss was related to the reduction in MA (*r* = −0.427, *P* = 0.008) and by multiple regression analysis independently associated with MA (*P* = 0.01). Thus, 11 patients in the dextran group (58 %) developed a clinical significant blood loss (>1500 ml) compared to only four patients (22 %) in the lactated Ringer’s group (*P* = 0.04).

**Conclusions:**

With the use of Dextran 70 vs. lactated Ringer’s solution during cystectomy, a relation between hemorrhage and coagulation competence is demonstrated. Significant bleeding develops based on an about 25 % reduction in thrombelastography determined maximal amplitude. A multivariable model including maximal amplitude discriminates patients with severe perioperative bleeding during cystectomy.

**Trial registration:**

The study was accepted on January 7^th^, 2013 at www.clinicaltrialsregister.eu EudraCT 2012-005040-20.

## Background

For patients exposed to a massive blood loss, maintained coagulation competence is important [1–3]. It is less clear whether coagulation competence influences bleeding during elective surgery. During surgery the circulation is supported by crystalloids, but about 30 % of the administered volume is “lost” to the interstitial space even when the circulating blood volume is reduced due to hemorrhage [4]. It therefore remains an option to administer a colloid to patients during major surgery because a colloid stays within the circulation and may even recruit fluid to the vasculature. Yet, it is a concern that synthetic colloids impair coagulation competence by reducing clot propagation and strength by affecting polymerization of fibrinogen [5–7].

We evaluated the importance of coagulation competence for the perioperative blood loss during cystectomy. The first part of this evaluation used Hydroxyethyl Starch (HES 130/0.4) vs. lactated Ringer’s solution (LR) and demonstrated a doubling of the perioperative blood loss [8]. Here we used Dextran 70 vs. LR to manipulate coagulation competence during cystectomy to evaluate whether the perioperative blood loss is related to reduction in coagulation competence as determined by thrombelastography (TEG). Also the design of the study allowed for comparison of the use of Dextran 70 and LR on patient outcome and the cost associated with administration of volume.

## Methods

This randomized controlled trial was based on plasma collected from participants in a colloid vs. crystalloid trial [8]. The study was approved by Rigshospitalet, University of Copenhagen, after approval had been obtained from the ethics committee in the Capital Region of Denmark (H-1-2012-135), and registered at www.clinicaltrialsregister.eu with Trial Registration Number EudraCT 2012-005040-20. The trial was monitored by the Agency for Good Clinical Practice at the University of Copenhagen and the Declaration of Helsinki criteria were followed [9].

Conduct of the trial and safety of the participants were overseen by the authors who gathered the data that remained confidential throughout the process. The authors were involved in all stages of manuscript generation and vouched for completeness and accuracy. No third party influenced the study design, data analysis, or reporting.

**Fig. 1:**
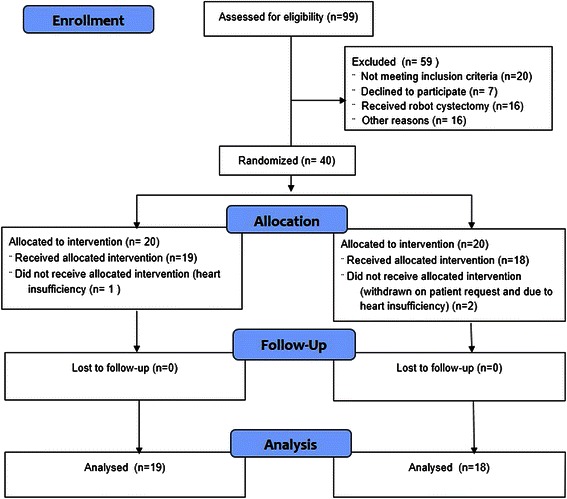
Randomization of Study Patients. Consort study flow diagram

Screening and randomization took place between February 12^th^ 2013 and July 8^th^ 2014. At least 24 h before surgery written informed consent was obtained from the patients. We excluded patients from this investigator-initiated, prospective, blinded trial if consent was withdrawn. Forty out of 99 patients scheduled for elective cystectomy were randomized to receive the recommended maximum volume (25 mL/kg) of either 6 % Dextran 70 (Macrodex®, Meda AB, Solna, Sweden) or LR by computer-generated allocation sequence (Fig. 1). We included patients older than 18 years. Scheduled for elective open cystectomy with no history of heart or hepatic insufficiency, disability of coagulation, intra-cerebral hemorrhage, or hemodialysis. If a patient used medication that was considered to affect coagulation competence, that medication was paused 5 days prior to surgery according to national guidelines.

### Interventions

The patients were allowed to eat solid foods up to 6 h and to drink clear fluids until 2 h before surgery. An IV line was established and flushed with the randomized fluid placed in an opaque bag to blind the surgical nurse who recorded the volume of lost blood. After induction of anesthesia a catheter was placed in the radial artery of the non-dominant arm and connected to a Nexfin monitor (Bmeye B.V, Amsterdam, The Netherlands). From the blood pressure curve, Nexfin estimates a beat-to-beat stroke volume (SV) and thereby cardiac output (CO) by use of a non-linear three-component model of arterial impedance as evaluated by modified Modelflow technology [10].

For induction of anesthesia remifentanil infusion (0.5 μg kg^−1^ min^−1^) was initiated and when the patient reported sedation propofol (2.0 mg kg^−1^) was administered. Propofol (5–10 mg kg^−1^ h^1^) and remifentanil (1.75–2.25 mg h^−1^) were used to maintain anesthesia. In the horizontally placed patient normovolemia was established, i.e. it was secured that after eventual repeated administration of 200 mL of the randomized fluid, SV increased by less than 10 % according to “goal directed fluid therapy” (GDT) criteria [11].

Heart rate (HR), mean arterial pressure (MAP), SV, and CO were determined after induction of anesthesia and insertion of the arterial catheter (*T*_*1*_), after resection of the urinary bladder (*T*_*2*_), at the end of surgery (*T*_*3*_), and 2 h thereafter in the recovery room (*T*_*4*_). If systolic pressure was below 80 mmHg, 5–10 mg of ephedrine was administered. Arterial blood was analysed by TEG: clot initiation (R-time), rate of development (Angle), formation (maximal amplitude, MA), and lysis after 30 min (Ly30) depicting hemostatic competence (TEG 5000, Haemoscope Corporation, Niles, IL) [12]. We also analysed blood for hemoglobin, creatinine, international normalised ratio (INR), platelets, dimer, and fibrinogen. Furthermore, blood was drawn from the central venous catheter for lactate and blood gas variables (ABL 825, Radiometer, Copenhagen, Denmark).

To avoid excessive administration of Dextran 70, both groups of patients received either LR or 5 % Human Albumin (HA) if considered in need by the anesthetist after infusion of the allocated fluid (non-study fluid). Fluid balance was determined at *T*_*2*_, *T*_*3*_, and *T*_*4*_. Two surgeons who together had completed more than 1100 cystectomies carried out the procedures.

The primary outcome variables were coagulation competence and the perioperative blood loss. We also compared the use of dextran or LR on patient outcome defined as a straight postoperative track: length of hospital stay ≤7 days without complications requiring treatment and on the cost associated with fluid administration to support the circulation.

### Cost-effectiveness analysis

The cost-effectiveness ratio is defined as the cost (C) per successfully treated patient (E), i.e., cost to keep a patient free of complications. The cost-effectiveness analysis used the difference in cost divided by the difference in benefit (C/E) [13] based on the 2015 price (Data-Ware-House ®, Rigshospitalet) for Dextran 70 (4.51 $ per 500 ml), LR (1.19 $ per 1000 ml), packed red blood cells (46.84 $ per 250 ml), and HA (15.60 $ per 250 ml).

#### Statistical methods

Patients were analysed in the group to which they were assigned (Table 1). The sample size calculation was based on data from a study comparing the effect on hemorrhage during cystectomy with the use of HES 130/0.4 vs. LR [8]. The blood loss was 2181 vs.1370 ml with a SD of 1190 vs. 603 ml [8]. The study was powered to detect a difference in blood loss of 800 ml between administration of Dextran 70 and LR with a two-sided alpha level of 0.05 and a power of 80 % for *t*-test with correction for multiple comparisons. Consequently, a sample size of 20 patients per group was included.

**Table 1:** Patient characteristics of the study groups

	Dextran (*n* = 19)	Lactated Ringer’s (*n* = 18)	*P* value
*Preoperative variables*			
Age – yrs.	68.1 (61.9–74.3)	67.8 (64.1–71.6)	0.94
Male – sex	15 (79 %)	12 (67 %)	0.48
Body weight (kg)	77.3 (56–99)	77.2 (52–110)	0.97
BMI – kg/m2	25.3 (24.1–26.8)	24.8 (23.7–28.3)	0.66
ASA – class	2.0 (1–3)	2.0 (1–3)	0.48
Cardiopulmonary disease	11 (58 %)	11 (61 %)	1.00
*Intraoperative variables*			
Anesthesia (min)	210 (198–275)	210 (211–260)	0.93
Blood loss >1500 ml	11 (58 %)	4 (22 %)	0.04
PRBCs	12 (63 %)	5 (28 %)	0.04
Ephedrine (mg)	16.7 (8.8–24.5)	29.2 (20.2–38.2)	0.03
*Postoperative variables*			
Straight track ^a^	4 (21 %)	11 (61 %)	0.02
Days until bowel movement	3.0 (1–7)	2.5 (2–30)	0.60
Complications	7 (37 %)	3 (17 %)	0.26
Re-operations	5 (26 %)	2 (11 %)	0.40
Hospital stay (days)	9.0 (5–24)	7.0 (6–92)	0.69

Values are either numbers (n), percentages (%), means or medians with 95 % confidence interval, or range. *P-*value by univariate analysis. BMI, body mass index; ASA class, American Society of Anesthesiologists, PRBC, packed red cells, ^a^ length of stay, ≤ 7 days without complications requiring treatment

The statistical analyses were performed before breaking the randomization code and data were analysed from the modified intention-to-treat population, defined as all randomly assigned patients except those who could be excluded without the risk of bias (Fig. 1). We used two-sided or unadjusted chi-square tests with odds ratio (OR) and t-test and Fisher’s exact test for continuous and dichotomous variables, respectively (SPSS version 20.0). Test for differences were performed using Pearson’s test, χ2 test for categorical data, and analysis of variance or Mann-Whitney *U-*test and Wilcoxon signed ranks test for continuous data when appropriate. Means and standard deviation (SD) or confidence interval (CI) are given, For possible determinants of perioperative bleeding, multiple logistic regression analysis defined variables from TEG and plasma hemostatic analyses that were independently associated with severe intraoperative bleeding (>1.5 l).

Also receiver operating characteristic (ROC) curves were used to predict perioperative bleeding. Areas under the ROC curve with the 95 % confidence Intervals (CI) were calculated. Optimal cut-points were obtained by minimizing the distance between the ROC curve and the upper left corner of the curve. Results are presented with 95 % CI and a two-sided *P* value <0.05 was considered to indicate statistical significance.

## Results

Patients were randomized into two groups (Fig. 1). There was no significant intergroup difference in baseline data including preoperative disease among patients (Table 1). After induction of anesthesia, five patients (26 %) in the colloid group were intravascular hypovolemic according to GDT criteria as compared to seven patients (39 %) in the crystalloid group, *P* = 0.495. In the colloid group patients received 24 (18 to 25) ml kg^−1^ Dextran 70 since three patients were considered to be hemodynamic stable before the intended volume (25 ml kg^−1^) was infused.

The perioperative blood loss was 2339 (1630 to 3048) ml in the colloid group and not significantly different in the LR group: 1822 (1160 to 2484) ml, *P* = 0.2 (Table 2). Yet, more patients in the dextran than in the LR group were exposed to clinical severe hemorrhage: 58 % vs. 22 % lost more than 1500 ml blood, OR = 4.8 (1.1 to 20.2), *P* = 0.04. In the LR group, one significant bleeding episode (5.5 l) was because of injury to the common iliac vein and in this case hemorrhage cannot be ascribed to coagulation competence. If that patient is eliminated from the analysis, the loss of blood was 2339 (1630 to 3048) ml vs. 1610 (1090 to 2130) ml in the dextran and LR group, respectively, *P* = 0.048. Table 2 also presents IV administration of study as well as non-study fluids. Transfusion was initiated at a hemoglobin of 4.9 mmol l^−1^ and the transfusion of packed red blood cells was 597 (302 to 893) vs. 313 (42 to 584) ml (*P* = 0.14) in the Dextran 70 and LR group, respectively. Thus, 12 patients (63 %) in the colloid group were provided with blood vs. five patient (28 %) in the crystalloid group, OR = 4.5 (1.1 to 17.9), *P* = 0.04. The fluid balance was positive by 1520 (1198 to 1842) ml in the dextran group and by 1925 (1580 to 2272) ml in the LR group (*P* = 0.03) also with more patients in the RL group having a positive fluid balance exceeding 1500 ml, 78 % vs. 21 %, OR = 13.1 (2.7 to 62.8) *P* = 0.001. The dextran group received 17 mg ephedrine and the LR somewhat more (29 mg, *P* = 0.03) resulting in no differences in MAP, SV or CO between the two groups of patients.

**Table 2:** Perioperative fluid administration, blood loss, and fluid balance

		Dextran (*n* = 19)	Lactated Ringer’s (*n* = 18)		*P* value
*Intravenous fluids*					
Lactated Ringer’s	987 ± 614	691–1283	2883 ± 690	2540–3226	
Dextran 70	1886 ± 355	1765–2006	–		
Packed red blood cells	597 ± 613	302–893	313 ± 545	42–684	0.14
HA	118 ± 210	17–220	519 ± 379	331–708	0.001
FFP	201 ± 362	0–251	102 ± 301	0–251	0.37
Platelets	92 ± 21	0–202	0 ± 0	0–0	0.10
*Total fluids balance*					
Total fluid infusion	4157 ± 1779	3300–5014	4121 ± 1352	3450–4794	0.95
Total fluid lost	2638 ± 1491	1918–3356	2196 ± 1380	1509–2881	0.29
Fluid balance	1520 ± 668	1198–1842	1925 ± 696	1580–2272	0.03
*Blood loss*					
Total blood loss	2339 ± 1470	1630–3048	1822 ± 1240	1160–2484	0.27

Fluid balance was calculated from the administered solutions, transfusion of blood products, urine output, and blood loss (suction and drainage) during anesthesia and in the recovery room. Blood products included packed red blood cells, fresh-frozen plasma (FFP), and platelets. Values are expressed as means with sd and 95 % confidence interval. *P-*values by univariate analysis

Table 3 presents results from TEG analyses. The lowest values of RT, Angle and MA in the analyses was observed in the dextran group at the end of anesthesia (*P* <0.001). Platelets values decreased in both groups during anesthesia (from 205 ± 69 to 141 ± 45 10^9^/l in the dextran group, *P* <0.001, and from 243 ± 65 to 198 ± 72 10^9^/l in the LR group) *P* <0.001, whereas dimer increased similarly in both groups (from 0.59 ± 0.50 to 1.08 ± 0.57 mg/l (dextran group) and from 0.38 ± 0.12 to 1.18 ± 0.65 μmol/ l (LR group); *P* <0.001). Hemoglobin decreased more (5.2 ± 0.6 vs. 6.1 ± 0.9 mmol/l) in the dextran group compared to the LR group, *P* <0.001.

**Table 3:** Comparison of the coagulation variables between the study groups

Variable	Dextran (*n* = 19)		Lactated Ringer’s (*n* = 18)		*P* value
Reaction time (min)					
T_1_	5.93	5.34–6.54	5.25	4.69–5.82	0.100
T_2_	5.85	5.34–6.36	4.70	4.19–5.22	0.002
T_3_	5.90	5.20–6.59	4.26**	3.83–4.70	0.001
T_4_	5.91	5.36–6.45	4.31*	3.74–4.89	0.001
Angle (°)					
T_1_	70.72	68.76–72.68	71.12	68.03–74.21	0.817
T_2_	60.87***	56.85–64.90	72.84	70.56–75.12	0,000
T_3_	54.92***	50.53–59.30	72.57	69.95–75.18	0,000
T_4_	58.92***	55.01–62.82	72.39	70.32–74.46	0,000
Maximal amplitude (mm)					
T_1_	65.17	61.93–68.42	63.98	60.25–67.72	0.614
T_2_	54.30***	49.53–59.08	64.39	61.39–67.40	0.001
T_3_	48.92***	44.65–53.19	62.08	58.9–65.27	0.000
T_4_	49.08***	44.83–53.34	63.11	52.33–59.09	0.000
Lysis after 30 min (%)					
T_1_	1.52	0.64–2.42	2.15	0.82–3.50	0.403
T_2_	1.17	0.31–2.04	1.69	0.57–2.80	0.444
T_3_	0.71	0.27–1.15	1.57	0.71–2.42	0.066
T_4_	1.05	0.21–1.89	2.30	0.63–3.97	0.146

TEG analysis after induction of anesthesia (T_1_); after resection of the urinary bladder (T_2_); at the end of surgery (T_3_), and 2 h thereafter in the recovery room (T_4_). Values are mean with 95 % confidence interval. *P-*value by univariate analysis

*P <0.05, **P <0.01 and ***P <0.001 difference from induction of anesthesia (T1) within the group

**Fig. 2:**
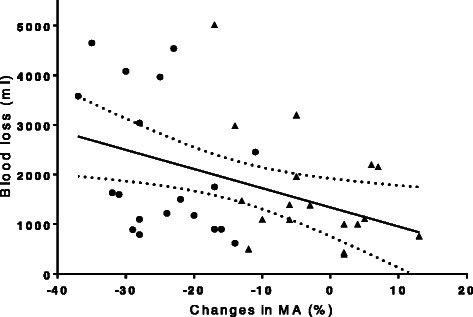
Maximal amplitude (MA) in relation to blood loss. Change in coagulation competence at the end of anesthesia expressed as MA by thrombelastography and blood loss in patients undergoing open radical cystectomy receiving either Dextran ● or lactated Ringer’s solution (RL) ▲(*n* = 37), *r* = −0.407 (95%CI −0.632 to −0.101), *P* = 0.008

**Fig. 3:**
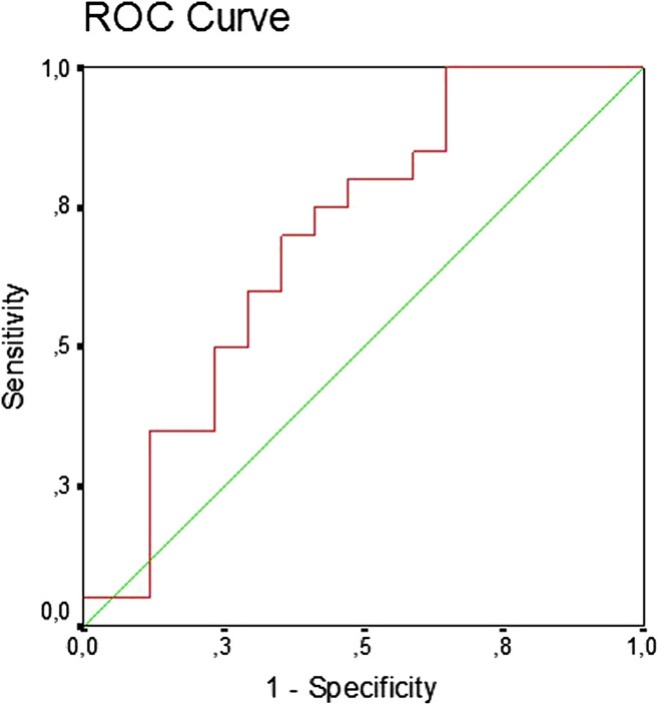
Receiver operator characteristic curve (ROC) for MA and probability of significant bleeding (>1.5 l). Receiver operator characteristic curves for MA in patients receiving Dextran or RL (*n* = 37) with blood loss >1.5 l (areal under the curve (AUC) 0.69 (0.51–0.87); the optimal cut-point for MA was 81 % of the value at T_1_, giving 80 % sensitivity and 53 % specificity (*P* = 0.04)

The blood loss was related to the decrease in MA (*r* = −0.407 (95%CI −0.632 to −0.101) *P* = 0.008 (Fig. 2), but not to R-time, Angle, or Ly30. Univariate analyses defined four TEG variables (Table 3) important for a blood loss >1.5 l and included in a multiple logistic regression analysis. Concerning the influence of the coagulation variables as predictors for what was considered a significant blood loss (>1.5 l), only MA was important (Table 4). Thus, MA was used to build a ROC curve for patients receiving Dextran 70 or RL with a blood loss >1.5 l. The areal under the curve (AUC) was 0.69 (0.51–0.87) and the optimal cut-point for MA was 81 % of the value at T_1_ (about 52 mm), providing 80 % sensitivity and 53 % specificity (*P* = 0.04) equating a likelihood ratio (sensitivity/1-specificity) = 1.90 (Fig. 3).

**Table 4:** Coagulation variables in predicting the development of severe blood loos during anesthesia

Variable	Regression coefficient (*β)*	Standard errors (SE)	*P-*value	Odds ratio (e^β^)	95 % CI
RT	0.61	0.86	0.48	1.84	0.34–9.95
Angle	−1.15	1.18	0.33	0.32	0.03–3.22
MA	2.48	1.25	0.04	11.88	1.03–136.9
Ly30	−0.37	0.84	0.66	0.69	0.13–3.62
Constant	−0.96	1.12			

*RT* reaction time, *Angle* angle in degrees, *MA* maximal amplitude, *Ly30* amplitude reduction after 30 min

Creatinine increased (>120 mol l^−1^) at the end of surgery for five patients (26 %) in the dextran group compared with normal values in the LR group (*P* = 0.046), but no participant was in need of hemodialysis. Also in the LR group 61 % of the patients had a postoperative straight track compared to only 21 % in the dextran group, OR = 5.9, 95%CI (1.38 to 25.23), *P* = 0.02 (Table 1). The number of patients with at least one treated postoperative complication was 7 (37 %) vs. 3 (17 %) (OR = 2.9, 95 % CI (0.6 to 13.8) *P* = 0.26). Five (26 %) vs. 2 (11 %) patients were re-operated in the dextran and LR group, respectively (OR = 2.9, 95 % CI (0.5 to 17.1) *P =* 0.40). No patient needed re-operation due to intestinal dysfunction. Thus, the length of hospital stay was similar among the two groups, *P* = 0.69.

The cost of fluid infusions per patient was119 vs. 95 $ (Dextran 70 vs. LR), while the cost-effectiveness ratio (the cost of successfully treating one patient with Dextran 70 or LR) was 188 (119 × 19 /12) and 114 (95 × 18/15) $ to avoid complications requiring treatment in the dextran and LR group of patients, respectively.

## Discussion

This randomised study used either Dextran 70 or lactated Ringer’s solution to support the circulation according to individualized goal-directed principles during cystectomy to evaluate the importance of coagulation competence for the perioperative loss of blood [14, 15]. Coagulation competence was evaluated by TEG and the perioperative blood loss was related to reduction in MA by 25 %.

Perioperative hemorrhage tended to increase when Dextran 70 rather than LR was administered and more patients in the dextran group were exposed to a severe blood loss as identified by a larger than a 1500 ml, blood loss (a class IV blood loss) and oxygen transport to the tissue might become affected [16]. Furthermore, three times as many patients receiving LR than dextran had a straight postoperative track supporting that Dextran 70 should not be the first choice colloid for support of the circulation during major surgery.

Dextran is used to support the circulation during major bleeding but obviously with moderate administration for minor surgery administration of Dextran affects coagulation competence only modestly [17]. In accordance with the CRISTAL trial addressing hypotensive patients under intensive care [18], the present investigation did not show significant difference in transfusion requirements between the two groups of patients. Nevertheless, the transfusion requirement tended to be larger with administration of Dextran 70.

LR reverses hypovolemia by plasma volume expansion [19]. Animal experiments and computer simulation based on fluid volume kinetics suggest that initial fluid volume resuscitation in a hypotensive patient may include administration of 600–750 ml of crystalloid fluid or 100 ml Dextran 70 [20]. Conversely, a positive postoperative fluid balance may provoke gut edema and contribute to intestinal dysfunction, postoperative complications, and extended hospital stay [21–23]. Thus it is a worry that the patients provided LR received almost 50 % more fluid, besides more inotrope medication compared those provided with dextran, but there appeared to be no difference in complication among the two group of patents.

If one treatment is more effective and less costly than another, that treatment is considered dominant and the use of LR seems to be the dominant strategy. Equally Kruer & Ensor argue that crystalloids are associated with greater effectiveness and lower cost compared with colloids in regard to morbidity [24].

We evaluated the predictive value of TEG variables for perioperative bleeding. Changes in MA in patients with blood loss >1.5 I provides the areal under the curve on 0.69 besides 80 % sensitivity and 53 % specificity. The optimal cut-point for MA was 81 % (52 mm) (Fig. 3) and MA was the best predictor (Table 4) to discriminate between blood loss >1.5 l or ≤1.5 l. In a prospective trial, 255 consecutive patients were studied to compare the ability of TEG (ROTEG™) to predict postoperative blood loss, and the angle was the best predictor of a blood loss >500 ml [25]. Yet, in a recent study comparing hydroxyethyl starch and lactated Ringer’s during cystectomy there was correlation between the perioperative blood loss and MA, with an optimal cut-point for MA on 86 % (54 mm)_,_ resulting in 88 % sensitivity and 69 % specificity [8].

There are limitations to this investigation: the design has a short observation period and a larger cohort is needed to evaluate an effect on survival or other major outcomes. Three patients were excluded from the trial after randomization (Fig. 1). Furthermore, in one patient hemorrhage seemed to be related to injury to the common iliac vein and one patient was hospitalized for an exceptionally long time, both events not necessarily related to the type of fluid administered. Furthermore, the different volume of albumin administration in the groups may interfere with outcomes such as hospital stay and postoperative complications.

## Conclusion

With the use of Dextran 70 vs. lactated Ringer’s solution during cystectomy it is demonstrated that there is a relation between hemorrhage and coagulation competence. During critical bleeding there is an about 25 % reduction in thrombelastography determined “Maximal Amplitude”, which discriminates those with severe perioperative bleeding. Also the design of the study allowed for demonstration of greater effectiveness and lower cost with the use of LR compared to Dextran 70 although the length of hospital stay was equal.

## Abbreviations

ASA: American Society of Anesthesiologists
BMI: Body mass index
FFP: Fresh-frozen plasma
GDT: Goal directed fluid therapy
HA: Human albumin
LR: Lactated Ringers’ solution
MA: Maximum amplitude
PRBC: Packed red blood cells
ROC: Receiver operating characteristic curves
RT: Reaction time
TEG: Thrombelastography
